# Spatiotemporal phase slip patterns for visual evoked potentials, covert object naming tasks, and insight moments extracted from 256 channel EEG recordings

**DOI:** 10.3389/fnint.2023.1087976

**Published:** 2023-06-13

**Authors:** Ceon Ramon, Uwe Graichen, Paolo Gargiulo, Frank Zanow, Thomas R. Knösche, Jens Haueisen

**Affiliations:** ^1^Department of Electrical and Computer Engineering, University of Washington, Seattle, WA, United States; ^2^Regional Epilepsy Center, Harborview Medical Center, University of Washington, Seattle, WA, United States; ^3^Department of Biostatistics and Data Science, Karl Landsteiner University of Health Sciences, Krems an der Donau, Austria; ^4^Institute of Biomedical and Neural Engineering, Reykjavik University, Reykjavik, Iceland; ^5^Department of Science, Landspitali University Hospital, Reykjavik, Iceland; ^6^ANT Neuro, Hengelo, Netherlands; ^7^Max Planck Institute for Human Cognitive and Neurosciences, Leipzig, Germany; ^8^Institute of Biomedical Engineering and Informatics, Technische Universität Ilmenau, Ilmenau, Germany

**Keywords:** EEG phase slips, phase jumps, cortical neurodynamics, VEP, cortical phase transitions, insight moments, eureka effects, visual object naming

## Abstract

Phase slips arise from state transitions of the coordinated activity of cortical neurons which can be extracted from the EEG data. The phase slip rates (PSRs) were studied from the high-density (256 channel) EEG data, sampled at 16.384 kHz, of five adult subjects during covert visual object naming tasks. Artifact-free data from 29 trials were averaged for each subject. The analysis was performed to look for phase slips in the theta (4–7 Hz), alpha (7–12 Hz), beta (12–30 Hz), and low gamma (30–49 Hz) bands. The phase was calculated with the Hilbert transform, then unwrapped and detrended to look for phase slip rates in a 1.0 ms wide stepping window with a step size of 0.06 ms. The spatiotemporal plots of the PSRs were made by using a montage layout of 256 equidistant electrode positions. The spatiotemporal profiles of EEG and PSRs during the stimulus and the first second of the post-stimulus period were examined in detail to study the visual evoked potentials and different stages of visual object recognition in the visual, language, and memory areas. It was found that the activity areas of PSRs were different as compared with EEG activity areas during the stimulus and post-stimulus periods. Different stages of the insight moments during the covert object naming tasks were examined from PSRs and it was found to be about 512 ± 21 ms for the ‘Eureka’ moment. Overall, these results indicate that information about the cortical phase transitions can be derived from the measured EEG data and can be used in a complementary fashion to study the cognitive behavior of the brain.

## 1. Introduction

This report, presents our findings on how spatiotemporal patterns of phase slip rates (PSRs) change and exhibit oscillatory behavior during covert visual object naming tasks. These phase slips are related to the phase transitions of the coordinated activity of cortical neurons and have been observed in microgrid ECoG of rabbits (Freeman and Barrie, 2000; Freeman and Rogers, 2002), microgrid ECoG data of a human subject who was a candidate for epileptic surgery (Freeman et al., 2006a), and in scalp EEG data (Freeman et al., 2003; Ruiz et al., 2010; Freeman, 2015; Ramon and Holmes, 2015, 2020; Ramon et al., 2018). A general procedure is to extract the phase from the EEG data by use of the Hilbert transform, unwrap it, and then one can see episodic phase shifts. After detrending the unwrapped phase, one can see sharp phase slips at the location of episodic phase shifts. In coordination with phase slips on nearby electrodes, these form spatial patterns, called phase cones which travel on the measurement surface, i.e., cortical surface for the ECoG data and scalp surface for the EEG data. One can observe the amplitude and phase modulated waves in theta (3–7 Hz) and alpha (7–12 Hz) bands on the cortical or scalp surface with carrier frequencies in the beta (12–30 Hz) and low gamma (30–50 Hz) bands (Freeman and Barrie, 2000; Freeman and Rogers, 2002; Freeman et al., 2006a; Ruiz et al., 2010; Kozma and Freeman, 2017). Aperiodic resetting of these phase waves at theta and alpha band frequencies has also been observed (Freeman and Barrie, 2000; Freeman et al., 2003; Kozma and Freeman, 2017). A common theme in all of these studies has been to observe the spatiotemporal behavior of phase slips extracted from the ECoG or EEG data. These phase slips are also often called phase jumps (Pikovsky et al., 2001).

The formation of these phase slips and related spatiotemporal phase waves have been predicted from theoretical models and measurements of self-organized criticality of cortical networks at small and large scales (Freeman and Vitiello, 2006, 2010; Thatcher et al., 2009, 2014; Beggs and Timme, 2012). At any given moment neurons are firing in a random fashion that gives rise to the bulk of scalp EEG which we commonly measure and use it to analyze various quantities, such as power spectral densities, phase synchronization, etc., during spontaneous and evoked potentials studies. In addition, within small regions (∼ 0.5 mm) of the cortex, the electrical activity of a group of neurons is close to the state of criticality (Freeman, 1994, 2008, 2015; Beggs and Timme, 2012; Bettinger, 2017; Kozma and Freeman, 2017) and any slight external input (e.g., visual stimuli) or an internal input (a thought) could cause a state transition which will cause these neurons to fire in a coordinated fashion (Freeman et al., 2006a,b; Freeman, 2015). This coordinated firing produces a short burst of oscillations (Wright, 2009; Freeman and Quiroga, 2013) that contribute toward the bulk of EEG activity and create small perturbations or glitches in the EEG data (Freeman and Rogers, 2002; Kozma and Freeman, 2017). This is also corroborated by simultaneous measurements from scalp and depth electrodes in patients for epilepsy surgeries that some features in scalp EEG measurements could be related to the observed details from depth electrodes (Ramantani et al., 2014, 2016). Of course, depth electrodes showed more details of the temporal resolution of peaks which were slightly subdued in the scalp EEG but recognizable.

The sizes of the domain of criticality in neuronal tissue samples or in the brain, in general, are at microscopic to mesoscopic scales (Buzsaki, 2006; Hughes and Crunelli, 2007; Beggs and Timme, 2012; Tognoli and Kelso, 2014). However, larger and more areas of criticality in the brain are possible, such as, in the case of visual evoked potential studies where several areas of the cortex are simultaneously or sequentially active (Freeman, 2015). Measurements at the cellular level show the presence of in-phase and anti-phase synchronization of neuronal assemblies at millisecond time frames and how action potentials are influenced by the local field potentials (Buzsaki, 2006; Hughes and Crunelli, 2007). One can also observe in these measurements, thalamocortical oscillations with theta (3–7 Hz) and alpha (7–12 Hz) rhythms (Hughes and Crunelli, 2007). This in-phase and anti-phase episodic shift can be considered as a high speed switch for a collection of neurons (Thatcher et al., 2014) and possibly, could be related to the rate of the state transition for this collection of neurons (Freeman, 1994; Freeman and Vitiello, 2006; Kozma and Freeman, 2017). Since ECoG and EEG arise from the cellular level local field potentials, one could expect to see these state transition related activities in the measured ECoG and EEG data sets.

When groups of neurons in these criticality domains go through a state transition, they act like foci to generate spatiotemporal phase waves on the neocortical surface (Freeman et al., 2006b; Wright, 2009, 2016; Freeman and Quiroga, 2013). When these phase waves spread, they tend to incorporate larger areas of the brain also. This would also suggest that it is possible to see cortical phase transition related signatures, i.e., phase slips, on single or multiple electrodes in ECoG and EEG recordings. This has been confirmed by observed episodic phase slips at the location of perturbations in the ECoG and EEG data (Freeman and Rogers, 2002; Ruiz et al., 2010; Myers et al., 2014; Freeman, 2015; Ramon et al., 2018). A positive phase slip or peak will represent the growing coordination of firing of neurons in the local neighborhood while a negative phase slip or valley will represent the loss of coordination between nearby neurons (Freeman, 2003). The state transition related phase slips are different from the phase synchronization between two EEG signals. These state transitions in the EEG literature are also often called phase transitions or EEG phase transitions.

The above described critical behavior of the cerebral cortex at small or large scales is very similar to the triple point of water at the boundary of solid, liquid, and gas phases. Another example of criticality will be the sandpile model of self-organized criticality (Bak et al., 1987) in which one keeps adding grains of sand to a sand pile till it collapses. There are many other examples of criticality and phase transitions in physics and biology which are very well summarized in a recent review paper (Heffern et al., 2021).

These phase slip techniques have been successfully applied to show the increase in phase cone turnover rates in the epileptogenic areas from the epileptiform-free interictal EEG data (Ramon et al., 2013; Ramon and Holmes, 2020) and also the formation of oscillatory patterns of phase cone formations near to epileptic spikes (Ramon et al., 2018). Similarly, these techniques have also been applied to study the dynamical formations of phase cones from the micro-ECoG data collected with an implanted 8 × 8 microgrid, 1.25 mm interelectrode separation, fixed onto the surface of the right inferior temporal gyrus of a subject going through epileptic presurgical evaluations (Freeman et al., 2006a). One can see the waxing and waning of phase cones over the microgrid. However, the application of these techniques to study human cognitive behavior is relatively new. We found one animal rabbit study on phase slips and phase cones related to hypothesized cognitive cycles in the emergence of awareness during trained visual stimuli experiments (Davis et al., 2013; Myers et al., 2014; Kozma and Freeman, 2017). In another study, global spatial formations of phase slips and phase cones in the beta (15–22 Hz) band were observed during visual-auditory conditioned stimuli from the 64-electrode EEG data (Ruiz et al., 2010). We did not find other studies relevant to the work presented in this report.

A detailed study of spatiotemporal patterns of phase slips from the scalp EEG data during covert visual object naming tasks is new and has not been reported before, to our knowledge. Given the specifics of our experiment, we also examined the spatiotemporal patterns and the duration of insight moments (Kounios et al., 2008; Myers et al., 2014) during the first second of the post-stimulus period. One can observe well-defined activity over memory and language areas in PSR plots during the different stages of insight moments. However, the spatial plots of PSR were slightly different from EEG plots suggesting that PSR maps pick up different areas of cortical activity related to phase transitions of the coordinated activity of cortical neurons. Our findings suggest that PSR analysis might be an important new tool to study spontaneous and evoked brain activity. Some background material on insight moments is given below.

### 1.1. Insight moments

Insight moments are very common and are also called, “Aha!” or “Eureka” moments. These relate to suddenly or spontaneously finding a solution to a problem. Well-known examples are Sir Isaac Newton getting inspired by a falling apple to develop the theory of gravity or the famous ‘Eureka’ moment of Archimedes. The ‘Eureka’ moment is part of the four stages of creativity suggested by Wallas (1926) which are commonly called: Preparation, Incubation, Illumination, and Verification (Wallas, 1926; Gell-Mann, 1994; Sadler-Smith, 2015). Illumination is the ‘Eureka’ moment.

Nowadays, these insight moments can be studied with scalp EEGs, either in the resting state or in evoked response potentials studies (Bowden and Jung-Beeman, 2007; Kounios et al., 2008; Kounios and Beeman, 2014; Salvi et al., 2015; Oh et al., 2020). A typical procedure is to show a picture of an object on the screen and ask the subject to press a button or overtly say when he/she recognizes the object while the stimulus, i.e., picture, is still displayed on the screen. In this protocol, the object recognition time is counted from the onset of the stimulus. In general, during the stimulus period, one can see well-defined prominent peaks related to various aspects of insight moments in the EEG data between 50–1,000 ms from the start of a stimulus. One example of peaks will be the P1, N1, and P2 within the first 300 ms of the stimulus period. A burst of alpha (7–12 Hz) power followed by similar changes in the low gamma (30–50 Hz) band related to insight moments has been observed (Kounios and Beeman, 2014).

Another protocol is to show the picture for a second or so on a computer monitor and then turn the picture off and the subject has been instructed to name the object covertly or overtly from the moment the stimulus is turned off. Many variations of these protocols have been used for visual object naming under various scenarios. Out of these, our focus is mainly on covert visual object naming during post-stimulus periods which have been used before on epilepsy patients for language and memory area localizations from EEG and ECoG data sets (Ojemann et al., 1989; Ramon et al., 2009; Abel et al., 2016; Ojemann, 2016). Recently, similar protocols have also been used with normal subjects for studying the differences between visual perception (during stimulus) and mental imagery during the post-stimulus period (Dijkstra et al., 2019; Proverbio et al., 2023).

Different stages of the insight moment and related cortical phase transitions, i.e., phase slip rates have not been previously examined in detail during the post-stimulus period. This is one of the foci of the present study. Response time during the post-stimulus period is generally counted from the start of the post-stimulus period (Freeman and Rogers, 2002; Myers et al., 2014; Kozma and Freeman, 2017; Proverbio et al., 2023) and not from the onset of the stimulus.

Insight moments have also been studied through mental imagery, i.e., visualization of an object during the resting state or meditation. No external stimulus is needed. With this method, EEG power spectral density changes, synchronization, and complexity measures have been observed (Luft et al., 2019; Furutani et al., 2020; Giannopulu et al., 2022). Mental imagery and visualization during the post-stimulus period are similar. Also, mental visualization and perception, i.e., looking at an object on the screen, do share some neural responses, particularly in the alpha band (Dijkstra et al., 2018, 2019; Xie et al., 2020). Thus, one can study insight stages both during the stimulus and/or post-stimulus period even though both protocols are different.

Details of methods, results, and discussions are given in the following sections. In the Methods section, details of human EEG data collection of five subjects, temporal and spatial filtering of the EEG data, and extraction of phase slips from the EEG data are described. In the Results section, the spatiotemporal structure of phase slips during covert visual object naming tasks is described. Some critical analyses of our results and possibilities of any artifacts in our procedures are described in the Discussion section.

## 2. Materials and methods

### 2.1. EEG data

The EEG data of five adult male subjects were collected at the Institute of Biomedical and Neural Engineering, Reykjavik University, Iceland, and at the Technical University, Ilmenau, Germany with the approved Human Subjects Guidelines at Reykjavik University. The data sets were collected with an ANT Neuro 256-channel system with a 16,384 Hz sampling frequency for each channel. A high sampling rate helps in better extraction of the time series of PSR in a shorter stepping window. More details about this are described later on in EEG Phase Slip Extraction. Data sets were collected with reference to the Cz electrode but re-referenced to the common average reference for data analysis. Data sets were de-identified and then used for the research.

### 2.2. Protocol details

The protocols were explained to subjects before the start of the data collection and no instructions were given during the data collection. A dry simulation run was done first to familiarize subjects with data collection procedures and after that, the actual experiment was performed. The protocol was to show the picture of an object for 1 s (stimulus period) on a computer monitor, and then the picture disappears from the monitor screen and remains blank. The response was collected for 10 s during the post-stimulus period. The subjects were instructed to immediately begin to covertly visualize and name the object after the picture disappears. Subjects’ eyes were open during the post-stimulus period. An audio beep was given at the end of the trial to cue them to get ready for the next trial. After a random delay of 1.0–5.0 s, the next picture appeared on the screen. The audio beep was 300 ms long coming out of the computer speaker. No headsets or earbuds were used to hear the audio. The total length of each trial was approximately 11–15 s.

The experiment was repeated with thirty different pictures. We used black line drawings on a white background of common objects, such as a pencil, chair, cat, etc., which have been used before (Ojemann et al., 1989; Ramon et al., 2009). The EEG data set was continuously collected during the experiment. After filtering and artifact removal, the first trial was discarded as it was considered to be a familiarization trial for the experiment. The remaining data from 29 trials were averaged for each subject.

In our protocols, we did not ask subjects to identify and name the object during the stimulus period. Thus, we cannot do the complete insight moment analysis from the stimulus period EEG. However, the P1-N1-P2 complex of visual evoked responses appears within the first 300 ms (−1.0 to −0.7 s) after the start of the stimulus and can be related to the initial stages of the insight moments. After that later insight stages would be difficult to ascertain because we did not ask subjects to identify and name the object during the stimulus period. Thus, during the stimulus period, we are limited to analyzing the P1-N1-P2 complex only.

### 2.3. Inter-trial and inter-subject variability

The inter-trial and inter-subject variability of data sets were checked. Our work is based on the phase slips, so the variability was checked on the phase of the EEG data. Filtering and preprocessing of the data were done first. These steps are described in the next section. The phase was extracted after taking the Hilbert transform of the data. The RMS (root mean square) power of the phase of the data for each trial was computed. With reference to the lowest value, the percentage change in the other 28 trials was computed. Their mean and standard deviation (mean ± std) over 29 trials for each subject were computed and are listed in Table 1. The mean of the percentage change over five subjects is 2.63 ± 2.43 (*n* = 5). These variabilities are reasonable. Subject #4 had the highest (6.9%) mean percentage change and it varied between the range of 2.8% to 11%. The highest value of 11% was only for four trials out of a total of 29 trials. Thus, in essence, the data sets do not have a large variability and should be considered reasonably accurate for further analysis.

**TABLE 1:** Inter-trial variations in the phase power.

Subject #	mean ± std (*n* = 29)
1	0.74 ± 0.4%
2	1.9 ± 0.9%
3	1.6 ± 1.5%
4	6.9 ± 4.1%
5	2.0 ± 0.90%

Mean of all subjects: 2.63 ± 2.43% (*n* = 5).

### 2.4. Filtering and preprocessing

The EEG data sets were referenced to the common average reference. The data sets were filtered in the 3–49 Hz passband with an equiripple filter and then filtered again with a spatial harmonic analysis (SPHARA) filter to remove spatial artifacts (Graichen et al., 2015). The upper frequency of 49 Hz was selected to eliminate the contamination from the power-line frequency of 50 Hz in Europe where the data sets were collected. The SPHARA filter extends the classical discrete spatial Fourier analysis to non-uniformly positioned sensors on a surface in three-dimensional space, e.g., EEG sensors on the surface of the head. It represents a generalization of the spatial Fourier analysis for sensor arrangement on arbitrary surfaces. Determining the basis functions of this approach is based on the Eigen analysis of the discrete Laplace-Beltrami operator that is defined on the triangular mesh describing the sensor setup (Graichen et al., 2015). In contrast to PCA (principal component analysis) and ICA (independent component analysis), which rely on measured data to determine the components, the SPHARA approach uses only information about the sensor topography to calculate the basis functions.

Using the SPHARA approach, spatial filters can be designed to remove a few certain types of artifacts. The EEG signal and certain classes of interference, e.g., uncorrelated sensor noise and single channel dropouts, exhibit different spatial-spectral signatures which can be utilized for designing of spatial filters. The main contribution to the energy of the potential distribution on the surface of the head is made by spatial low-frequency basis functions. This is caused by the lowpass properties of the volume conductor model of the head (Srinivasan et al., 1998; Nunez and Srinivasan, 2006; Ramon et al., 2006). In contrast, uncorrelated sensor noise and single-channel dropouts show a flat spatial spectrum. By selecting a suitable spatial cutoff frequency for the filter, a large part of the energy of the noise can be suppressed and at the same time, a substantial part of the energy of the wanted signal is retained. The SPHARA approach is particularly suitable as a pre-processing step for subsequent phase analysis methods since the individual time samples are filtered independently of each other. The phase information in the time domain is not changed by SPHARA-based filtering.

**FIGURE 1:**
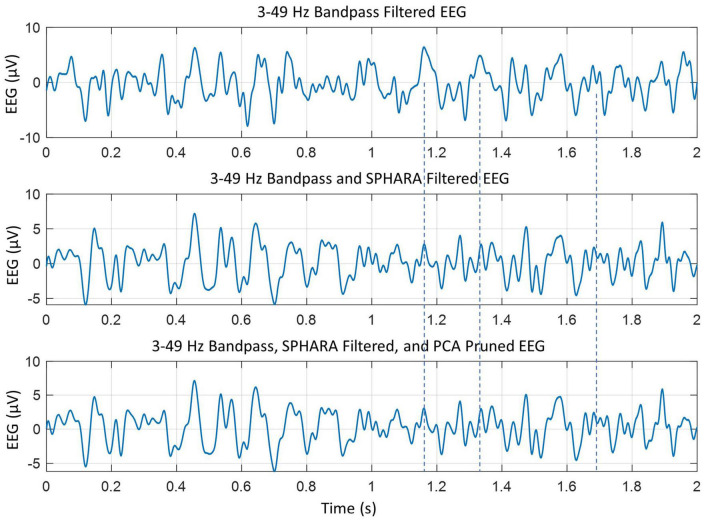
The effects of SPHARA filtering and PCA pruning of the bandpass filtered EEG data. The individual peaks become more visible in the **middle** and the **bottom** plots as compared to the **top** plot. This is more extenuated during the 1.0–1.8 s time frame. For example, look at the peaks near the vertical dashed lines.

Further artifact removal was performed with principal component analysis (PCA) using the EEGLAB software. With a combination of SPHARA and PCA, we were able to remove eye blinks, muscle artifacts, and cardiac (ECG) artifacts. An example of filtering effects is given in Figure 1. The SPHARA filtering and PCA pruning enhance the resolution of the peaks. For example, compare peaks in three plots between the duration of 1.0–1.8 s. In particular, look at the peaks identified by vertical dashed lines. In the middle and the bottom plots, several peaks are visible but obscured or merged in a broad peak in the top plot. This shows that the SPHARA filter and PCA pruning help to reduce the artifacts and enhance the resolution of peaks in the EEG data. The bottom two plots have only minor differences, on the order of about 10% or less. Also, the changes in peak values are approximately ± 0.5 μV or less. This would suggest that the SPHARA filter, as predicted (Graichen et al., 2015), is capable of removing most of the artifacts in the EEG data. The PCA pruning was done again, and no components were found that needed to be removed. The cleaned data of all subjects was rechecked again manually and with EEGLAB software to ensure that there were no artifacts, and none were found.

### 2.5. EEG phase slip extraction

**FIGURE 2:**
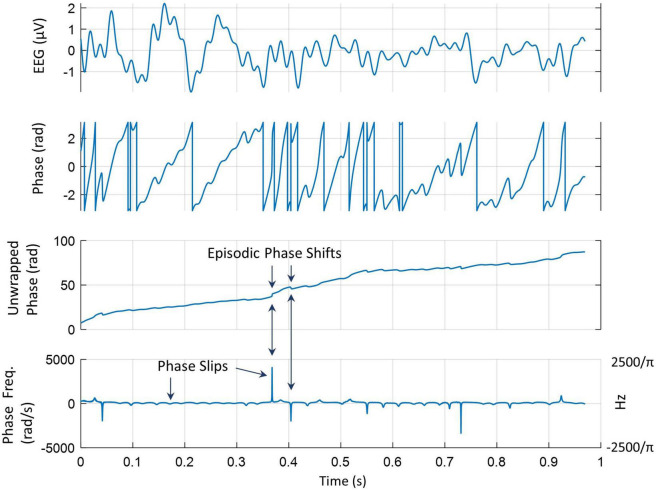
A pictorial representation of phase slip extractions. **(Top)** A 1-s-long EEG trace from one of the electrodes; **(second from the top)** sawtooth pattern of the phase extracted after taking the Hilbert transform of the EEG data; **(third from the top)** unwrapped phase of the EEG data; **(bottom)** derivative of the unwrapped phase divided by 2π gives the phase frequency in Hz. It shows sharp positive and negative phase slips.

All procedures for phase slip extraction from EEG or ECoG are described in detail in previous studies (Freeman and Rogers, 2002; Freeman, 2004; Ruiz et al., 2010). Similar procedures are being used here. The phase was extracted from the EEG data by use of the Hilbert transform and then it was unwrapped to give almost a linearly increasing time series of the phase. The linear trend in the unwrapped phase was removed by doing the first-order differencing or derivative (*d/dt*) of the unwrapped phase. These procedures are described in Figure 2. The top plot is a 1-s-long EEG trace from one of the electrodes. The second plot from the top is the sawtooth pattern of the phase extracted after taking the Hilbert transform of the EEG data. The unwrapped phase in radians is given in the third plot from the top which has an approximately linearly increasing phase with time. In addition, it shows episodic phase shifts in the unwrapped phase. The bottom plot is for the phase frequencies. These were obtained by taking the derivative of the unwrapped phase and dividing by 2π giving us the phase frequency in cycles/s or Hz. There are sharp positive and negative peaks which are called phase slips. A positive phase slip will create a growing spatial phase cone structure that has a potential to spread on the scalp or cortical surface while a negative phase slip represents a collapsing spatial phase cone structure on the scalp or cortical surface (Freeman and Quiroga, 2013; Kozma and Freeman, 2017).

The application of the Hilbert transform to EEG data will also generate these phase slips from random noise activity. To separate from the random noise, one needs to incorporate the biological and electrophysiological information regarding the firing of neurons and the formation of phase slips (Freeman et al., 2006a; Ruiz et al., 2010; Ramon and Holmes, 2015, 2020; Ramon et al., 2018). These include: (1) phase slip frequency is within a given temporal band, e.g., 7–12 Hz for the alpha band, (2) sign of the positive or negative peaks did not change for at least two consecutive time steps, and (3) the magnitudes of the two consecutive peaks were the same within the limit of ± 0.01. As an example, for 10 Hz, it will be between 9.99 to 10.01 Hz. Application of these criteria will significantly reduce or eliminate the counting of phase slips due to random noise and will produce a reasonable continuous time series of PSR. These criteria are based on previous studies (Ruiz et al., 2010; Ramon et al., 2013, 2018).

The PSR (counts/ms) was computed with a stepping window of approximately 1.0 ms width and with the step size of one digitization point, i.e., 0.061 ms which is based on the sampling rate of 16,384 Hz of the EEG data. The actual window size was 16 digitization points, that is (16/16,384) = 0.976 ms. Hereafter, for simplicity, we will call it a 1.0 ms window. The step size should be 5–10% of the window size to reduce temporal smearing and aliasing errors due to the undersampling of the data. Thus, a step size of one digitization point, 0.061 ms, is a good choice for a stepping window of 1.0 ms.

A higher sampling rate helps in better analysis of the spatiotemporal spread of the EEG and the derived quantities, such as PSR, from time series data. Keeping the stepping window size small, i.e., 1 ms, also helps in reducing the temporal and spatial smearing of the PSR. The 1.0 ms window is still narrow enough to cover the depolarization (∼1.0 ms), repolarization (∼1.0 ms), and the refractory (∼ 2 ms) period of the action potential with an adequate temporal resolution (Nordlie et al., 2010). At a lower sampling rate, say at 1 kHz, the window size needs to be increased to about 10 ms or so to get any reasonable computations of the PSR. This will smear the computed PSR significantly, which might not correctly incorporate the electrophysiological information in the computed time series of the PSR. Another point to note is that in earlier experimental measurements it was found that spatial phase cone gradients propagate on the cortical surface with velocities up to 2 mm/ms (Barrie et al., 1996; Freeman et al., 2003). Also, the range of conduction velocities of cortical axons is in the range of 1–10 mm/ms (Budd and Kisvárday, 2012; Swadlow and Waxman, 2012). Thus, a high sampling rate will help to capture finer details of these phase slips and their related spatiotemporal phase waves on the cortical surface and the scalp. Once the time series of PSR has been extracted from the EEG data, then one can resample it at a lower rate for any subsequent further analysis.

In summary, the pipeline used for computing PSR consists of these steps: (1) Filter EEG in the appropriate EEG band, e.g., 7–12 Hz for the alpha band, (2) Extract phase slips based on the criteria described above, and (3) Compute phase slip rate (PSR) in 1.0 ms wide stepping window with a step size of one digitization point, ∼0.06 ms.

**FIGURE 3:**
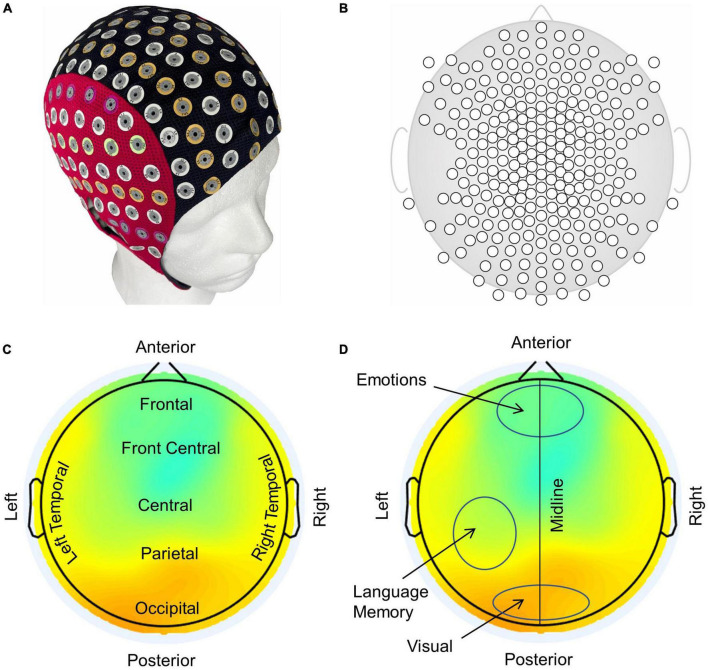
**(A)** A 256-electrode cap on a phantom head, **(B)** electrode layout on a flat surface, **(C)** anatomical nomenclature used in identifying different areas of the brain in EEG plots, and **(D)** prominent functional areas of the brain associated with scalp EEG plots.

Spatiotemporal plots of the phase slips and the EEG potentials were made with a montage layout of 256 electrode positions on a flat surface using the EEGLAB software (Delorme and Makeig, 2004). A smoothing spline curve fitting with a smoothing parameter of 0.99995 was used for spatiotemporal plotting of the computed PSR data. A typical example of a 256 EEG electrode system and its electrode layout is given in Figure 3. The electrode cap on a phantom head (Fiedler et al., 2022) is shown in Figure 3A, followed by the electrode layout on a flat surface in Figure 3B. A typical example of the EEG potentials is shown in Figure 3C which also shows typical names, e.g., frontal, parietal, temporal, etc., to describe the spatial locations in the plot. Approximate locations of visual, language, memory, and emotional areas are marked in Figure 3D which roughly correspond to the respective activation areas in the brain areas (Gazzaniga and Mangun, 2014; Dixon et al., 2017). The activity in the visual area is at the bottom (posterior) of the plot which is roughly above the occipital lobe of the brain. The electrodes at the top (anterior) of the plot pick up the emotional responses from the frontal lobe. The language and the memory areas in the left temporal and parietal lobes and their activities will be picked up by the electrodes in the left central-parietal areas. However, memory areas are also distributed in other parts of the brain. The midline separates the left and right sides of the scalp. From these plots, one can infer the cognitive behavior of the brain under various conditions, including visual object-naming tasks.

Most of the data analysis was performed on a desktop computer with an 8-core CPU and 112 GB of memory. The majority of work was performed using EEGLAB (Delorme and Makeig, 2004) software in the MATLAB environment and, in addition, several software codes were written for phase slip analysis, post-processing, and visualizations of the computed results. For analyses of data sets requiring larger (>112 GB) memory, we used MATLAB software at the UCSD (University of California, San Diego) supercomputing center using their NSG (Neuroscience Gateway) portal.

### 2.6. Surrogate data

Surrogate data testing was performed to make sure that our results are real and are not due to chance. Procedures for performing this analysis are described elsewhere (Prichard, 1994; Kugiumtzis, 2002; Lancaster et al., 2018). The simplest procedure is to generate random data from a subset of real data and perform exactly the same analysis on both data sets. In other words, filtering (Widmann and Schröger, 2012), differentiating, Hilbert transform application, phase slip extraction procedures, etc., should be the same for the real data and the surrogate data. Otherwise, one could get spurious results (Prichard, 1994). If the results for the real and the surrogate data are significantly different, then the phenomena or process under investigation is genuine and not a chance occurrence.

For our work, the surrogate data was generated by randomly shuffling the real data of one of the subjects. A 5-s-long filtered EEG data of one subject, as described above, was randomly shuffled using the ‘randperm’ command in MATLAB, and its power spectral density was calculated and checked to make sure that it was different from the real data and very close to being a white noise. This shuffled data was then used to compute the rate of formation of phase slips (counts/ms) over 5 s in the frequency band of 3–49 Hz. This process was repeated for 100 trials. The average value of the rate of phase slips over 100 trials was: 0.2 ± 0.002 (*n* = 100) counts/ms. This value (0.2 counts/ms) is a very small number and much less than the phase slips of actual object naming experiments which were in the range of 0.0–15.0 counts/ms. Anything above 0.2 counts/ms should be considered as arising from biological processes and not from random noise. These values for randomly shuffled data are for reference only and have not been subtracted in all plots given in the Results section.

## 3. Results

### 3.1. Data analysis of subject #1

A detailed report of results for Subject #1 is given below. It includes: (1) time series profiles of EEG and PSR on two electrodes, (2) spatiotemporal profiles of EEG and PSR of three peaks, viz., P1, N1, and N2 in the stimulus period, (3) spatiotemporal profiles during the first second of the post-stimulus period, and (4) analysis of different stages of insight moments during the post-stimulus period.

#### 3.1.1. Phase slips of two electrodes for subject #1

**FIGURE 4:**
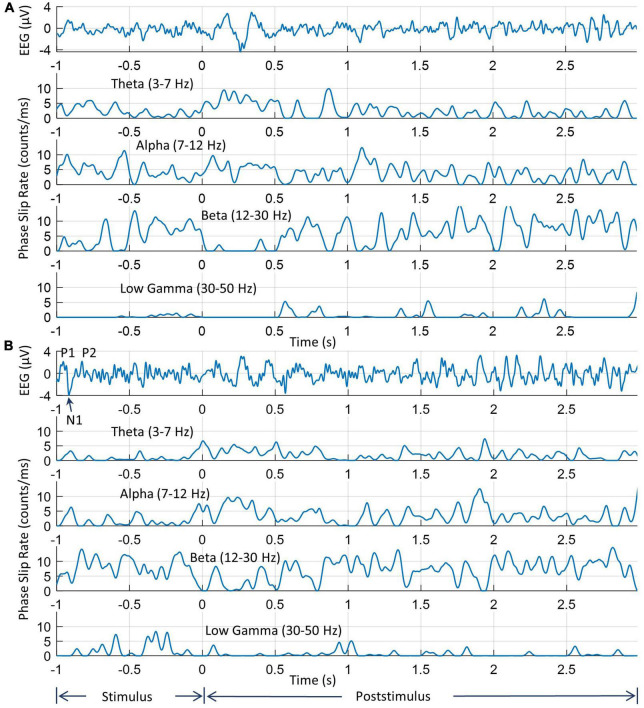
An example of EEG and PSRs in all four bands for two electrodes. **(A)** EEG and PSR from one of the electrodes near to left central midline area; **(B)** the same information from one of the electrodes in the left visual area. Note that time series profiles of PSRs at two electrodes are significantly different during the 0.0–1.0 s period when the subject is visualizing and analyzing the image of the object during the covert object naming tasks.

The EEG and PSR activities during the stimulus and post-stimulus periods on two electrodes are shown in Figure 4. Here the stimulus period is from −1.0 to 0.0 s when the picture was displayed on the computer monitor and the post-stimulus period starts at 0.0 s when the picture on the screen has been turned off and the subject begins to covertly visualize and name the object. The EEG trace and phase slips from one of the electrodes from the left midline central area which is involved in language and memory processing are given in Figure 4A. Similar information from one of the electrodes in the left visual area is given in Figure 4B. The theta band EEG activity is visible in both electrodes. Superimposed on large theta band oscillations are smaller oscillations that are related to alpha, beta, and low gamma bands.

**FIGURE 5:**
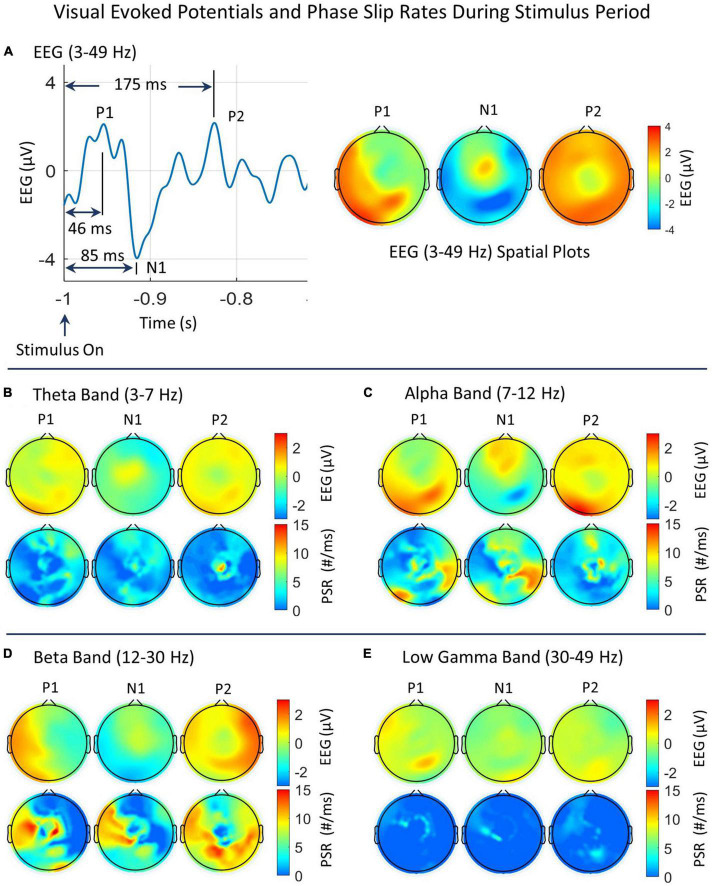
Spatial plots of EEG and PSRs during the stimulus period in different EEG bands. **(A)** Time series plot of EEG (3–49 Hz) from one of the electrodes in the left visual area. The prominent VEP peaks, P1, N1, and P2 are marked and their spatial plots are included. The P1, N1, and P2 peaks are located at 46 ms, 85 ms, and 175 ms, respectively from the start of the stimulus. **(B–E)** Spatial plots of EEG and PSRs in theta, alpha, beta, and low gamma bands. In each figure, the top row is for the EEG and the bottom row is for PSRs. The EEG is in μV and the PSR is in counts/ms.

The peaks of visual evoked potentials (VEP), P1, N1, and P2 during the stimulus period are marked in the EEG plot (Woodman, 2010). The locations of these peaks depend on the latency, i.e., the delay between the start of the stimulus and brain response, which varies from subject to subject (Makeig et al., 1999). For this particular subject, these P1, N1, and P2 peaks are located at 46 ms, 85 ms, and 175 ms, respectively, from the start of the trial at −1.0 s. Or as shown in Figures 4, 5, on the time axis of −1.0 to 0.0, the peak P1 is located at −0.954 s, the peak N1 is located at −0.915 s, and the peak P2 is located at −0.825 s, respectively. These EEG peaks during the stimulus period, are related to the initial evoked response of the visual, language, and memory areas in the brain. These are different from the knowledge analysis during the post-stimulus period where one is intentionally recognizing and analyzing a stimulus, i.e., an object in this study during the covert object naming tasks. The EEG activity on both electrodes begins to return to the normal resting state EEG patterns around 1.8 s from the start of the post-stimulus period at 0.0 s. The same patterns are also present in the PSR activity.

The PSRs (counts/ms or #/ms) for all four bands are also included for both electrodes. Overall, the low gamma band activity is subdued as compared with oscillatory activities in the theta, alpha, and beta bands. The peaks in the 0.0–1.0 s period are related to the covert visual object naming tasks during the knowledge cycle. These phase slip rates also exhibit the formation of amplitude and phase-modulated waves in the theta and alpha bands as has been predicted earlier based on the periodic resetting of phase transitions in the cortical neurodynamics (Freeman and Vitiello, 2006, 2010; Ruiz et al., 2010; Freeman, 2015). The EEG amplitude and the magnitude of PSR need not have a one-to-one correspondence with each other. The reason for this is that the phase slips are related to small perturbations in the EEG data which could be anywhere in the time series of the EEG data. For example, these could be at the high amplitude, low amplitude, or even near the zero crossings in the time series of EEG data. Similarly, PSR in a 1.0 ms wide window is not based on the amplitude but rather on the number of episodic phase shifts in the unwrapped phase which gives rise to the phase slips. Also for the same reasons, the PSR values are different in different EEG bands because the EEG profiles are different in theta, alpha, beta, and low gamma bands.

#### 3.1.2. Stimulus period for subject #1

As described earlier, there are three prominent peaks, P1, N1, and P2 during the stimulus period. A time series plot of EEG (3–49 Hz) from one of the electrodes in the left visual area is included in Figure 5A. The prominent VEP peaks, P1, N1, and P2 are marked, and their spatial EEG (3–49 Hz) plots are also included. At the P1 peak, most of the activity extends from the left temporal to the parietal and visual areas (back of the head). At the N1 peak, the positive EEG potential activity is focused in the midline central area and the negative EEG potentials are spread in the visual area. At the P2 peak, the activity is spread over the whole topography except at the central midline area. The breakdowns of the EEG and PSRs in different EEG bands at these peaks are given in additional plots, B, C, D, and E in Figure 5. In each plot, the top row is for the EEG and the bottom row is for the PSR. The EEG activity of the positive P1 peak in Figure 5A has major contributions from alpha, beta, and low gamma bands. The positive N1 peak has a major contribution from theta, alpha, and beta bands. The negative N1 peak has major contributions from the alpha and beta bands. The P2 peak has major contributions from theta, alpha, and beta bands.

In the theta band (refer to Figure 5B), The low level (∼ 1 μV) EEG activity at the P1 peak is mainly in the right frontal, temporal, parietal, and posterior (back of the head) visual areas. The positive (>0.0 μV) EEG activity at the N1 peak is mainly in the midline central area, and for the P2 peak, it is spread all over the topography except in the central midline area. The PSR activity in the theta band (Figure 5B) is remarkably different from the EEG activity. At the P1 peak, the PSR activity is in the frontal, right temporal, central, and parietal areas. There is some activity in the central and right visual areas which is slightly different from the EEG activity. The PSR is in the range of 0–7 counts/ms. For the N1 peak, the PSR activity is distributed in the frontal, central area, right front temporal, and visual areas (back of the head). In contrast, the EEG activity for the N1 peak in the theta band is very focused in the midline central area. For the P2 peak, in the theta band, the EEG activity is spread over the whole head with low (∼ 0.0–1.5 μV) activity at the central midline area. In contrast, the PSR activity has a bright spot (∼11 counts/ms) at the central midline area and some low-level (2–5 counts/ms) activity in the right front central area.

In the alpha band (refer to Figure 5C), the EEG activity goes through rapid changes in going from P1 to N1 to P2 which has a time gap of only 129 ms between P1 and P2. It starts with the visual and parietal areas at the back of the head to the frontal area at N1 and then spreads all over the head at P2. In contrast, the PSR activity at the P1 peak is in the left visual and right temporal-parietal areas. At the N1 peak, the PSR activity is in the frontal and right temporal areas. At the P2 peak, it has hot spots in the front central and the midline central areas. This contrast in spatial plots of EEG and PSRs could be due to event-related phase reorganizations (Klimesch et al., 2007; Fellinger et al., 2011). The negative hot spot in the N1 plot is in the right central parietal area which matches well with the high (∼ 12 #/ms) PSR activity right parietal area.

In the beta band (refer to Figure 5D), the positive EEG activity at P1 is mainly in the left temporal and visual areas, at N1 there is very low-level activity at the central midline area, and then at P2, it is distributed all over with stronger activity areas in the right temporal, right parietal, and frontal areas. The negative activity area for P1 is the right scalp area and for N1 it is in the posterior and temporal areas. In contrast, the PSR activity in the beta band at the P1 peak is high in the left temporal and central areas, at the N1 peak in the same areas on the left side, and then at the P2 peak, it is widely distributed at parietal and left and right temporal areas.

In the low gamma band (refer to Figure 5E), the EEG and PSR activities both are of low magnitude. At the P1 peak, the positive EEG activity is in the left front temporal and right parietal areas. The PSR activity is in the left frontal and right central areas with some correlations with EEG activity. At the N1 peak, the PSR activity is focused in the left central and temporal areas. This pattern changes at the P2 peak. The PSR activity at a low level (∼ 2–4 counts/ms) spreads in the left frontal and central areas and right parietal areas. There is some correlation with the EEG activity at the P2 peak.

A four-way ANOVA analysis was performed on the PSR values of all 256 electrodes at P1, N1, P2, and the PSR values at the beginning of the stimulus period at −1.0 s for each band separately. It was found that values were significantly (*p* < 0.01) different from each other.

These findings are very similar to what has been seen before for the visual evoked potentials (Mangun, 1995). The P1 activity is observed over the occipital (back of the head) area and its sources are expected to be related to the activation of the visual cortex. This component is related to visual attention (Mangun, 1995). The positive N1 component activation is mostly observed in the fronto-central area of the midline as we are seeing in Figure 5C. The companion negative N1 activation area is in the right central parietal area. The P2 activation area is generally in the centro-frontal and the parieto-occipital areas of the scalp and it represents some aspect of higher-order perceptual processing, modulated by attention (Backer et al., 2019).

#### 3.1.3. Post-stimulus period for subject #1

**FIGURE 6:**
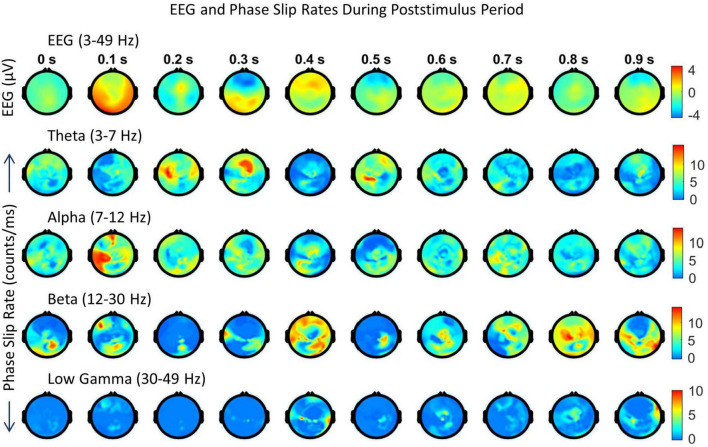
Spatiotemporal plots of EEG (μV) in the 3–49 Hz band and PSR (counts/ms) during the first second of the post-stimulus period. The spatial plots are at specific time points, e.g., 0.0 s, 0.1 s, etc. The temporal separation between the frames is 100 ms. The first row is EEG, followed by PSRs in the theta, alpha, beta, and low gamma bands. The EEG data sets were averaged over 29 trials and the PSRs were extracted from the averaged EEG.

In Figure 6, spatiotemporal plots of EEG and phase slips in all four bands are given during the post-stimulus period of 0–0.9 s with a temporal resolution of 100 ms which is sufficient to analyze the spatial changes related to visual, language, and memory processes. These spatial plots are at 0.0 s, 0.1 s, 0.2 s, etc., and data was not averaged between the frames. The EEG activity in the 3–49 Hz band is in the top row followed by the PSR spatial plots in theta, alpha, beta, and low gamma bands, respectively. The color-coded scales are given on the right side of the figure. The period of 0.0–0.5 s is considered to be the critical period of brain activity during which the consciousness is capturing the initial impressions of the image of the object in the visual cortex and recalling its name and form, i.e., shape, from the language and memory areas during the covert visual object naming tasks. This, in a way, is similar to the visual evoked potentials during the stimulus period (Kounios et al., 2008; Daliri et al., 2013) and in the analysis of insight moments during the post-stimulus period (Davis et al., 2013; Myers et al., 2014).

Looking at Figure 6, the positive EEG activity peaks at different locations during the period of 0.0 to 0.9 s. The prominent activity areas are: (1) at 0.1 s in the visual area (occipital lobes) at the back of the head, and also in the left and right temporal areas, (2) at 0.2 s in the front central midline area, (3) between 0.3 and 0.4 s, a major spatial shift from visual to the frontal area, (4) between 0.5 and 0.6 s, in most of the parietal and right areas, and (5) then slowly spreading and shifting in different parts of the topography during 0.7 to 0.9 s. The first spread at 0.1 s is the first wave observed during the covert visual object naming task (Myers et al., 2014) which is similar to the P1 wave during the stimulus period (Kounios et al., 2008; Daliri et al., 2013). The negative EEG potentials are very predominant in the frontal area at 0.3 s which matches with strong PSR activity in the theta band.

In comparison to EEG, the PSR activity is very different in the theta band. There is a hot spot in the left front central area at 0.0 s and then PSR decreases in subsequent frames till 0.2 when there is widespread activity in left and right centro-temporal areas. At the 0.3 s frame, it has a bright spot in the front central area and then activity gradually decreases in subsequent frames till 0.5 s where there is activity in the left parietal and right frontal areas. After that PSR at a low level, 3–7 counts/ms, spreads in several areas of the topography and then becomes mainly focused in frontal areas at 0.9 s.

The alpha band PSR spatial plots are given in the third row from the top in Figure 6. Main activity areas are in the left parietal and frontal areas at 0.1 s, then go through changes till 0.3 s where it is at a low level. After those hot spots in the left parietal and central areas at 0.4 s. Then there are several distributed hot spots at 0.7 s in the left visual, left central temporal (near to the left ear) areas, and also some activity in the right frontal area. During the period of 0.8 s to 0.9 s, the PSR activity is at a very low level (0–5 counts/ms) all over the topography.

The PSR activity in the beta band (Figure 6, fourth row from the top) at 0.0 s has a bright spot (∼ 12 counts/ms) in the right central parietal area and a low level (∼ 2–6 counts/ms) widespread activity in left temporal and central parietal areas. At 0.1 s, these activity areas become focused at left the frontal and central parietal areas which partially correlate with the EEG activity at 0.1 s. At 0.2 s, the PSR activity has almost receded to the right central parietal and occipital areas. This pattern changes at 0.3 s with a hot spot (∼ 12 counts/ms) in the left central temporal area and wider distributed activity in the right parietal area. Now moving on to the period of 0.4–0.7 s. The spread of the beta band PSR from 0.3 s to 0.4 s is very pronounced in the frontal, left front temporal, and right parietal areas. This correlates well with EEG activity at 0.4 s in the frontal areas only. There are several bright (∼ 7 counts/ms) spots at 0.7 s in the left front temporal, right temporal, and right parietal areas which have a slight correlation with the EEG activity. During the period of 0.8–0.9 s, the EEG activity shifts from central to right and left parietal areas. This probably signifies that EEG brain activity is moving from covert cognitive object-naming tasks to normal background brain activity. In contrast, the PSR activity in the beta band is very focused during this period. There are several hot spots in the central area at 0.8 s which become more confined in parietal areas at 0.9 s.

The low gamma band PSR activity (Figure 6, bottom row) is low during the 0.0–0.3 s period which changes to some low-level activity (∼ 3–6 counts/ms) at 0.4 s in the front central area, central area, and right parietal temporal areas. It has some similarities with the EEG plot at 0.4 s. After that, PSR remains low (0–5 counts/ms) during the period of 0.5 to 0.7 s with the majority of the activity in the central midline area. At 0.8 s, there is some activity (∼ 3–5 counts/ms) in the midline central area which spreads to left and right temporal areas at 0.9 s. Overall, it seems that PSR activities are beginning to return to the normal background activity after 0.8 s.

A paired Student’s *t*-test analysis was performed on the mean values of PSR for the 1-s-long data during the stimulus (−1.0 to 0.0 s) period and the same length (0.0 to + 1.0 s) of data during the post-stimulus period. We get 256 values, one for each electrode for the stimulus and post-stimulus period, respectively, and the Student’s *t*-test was performed on these two sets of values. This was performed for each band separately. Pre and post-stimulus values were significantly (*p* < 0.01) different for each band.

#### 3.1.4. Insight moment analysis for subject #1 during covert object naming tasks

**FIGURE 7:**
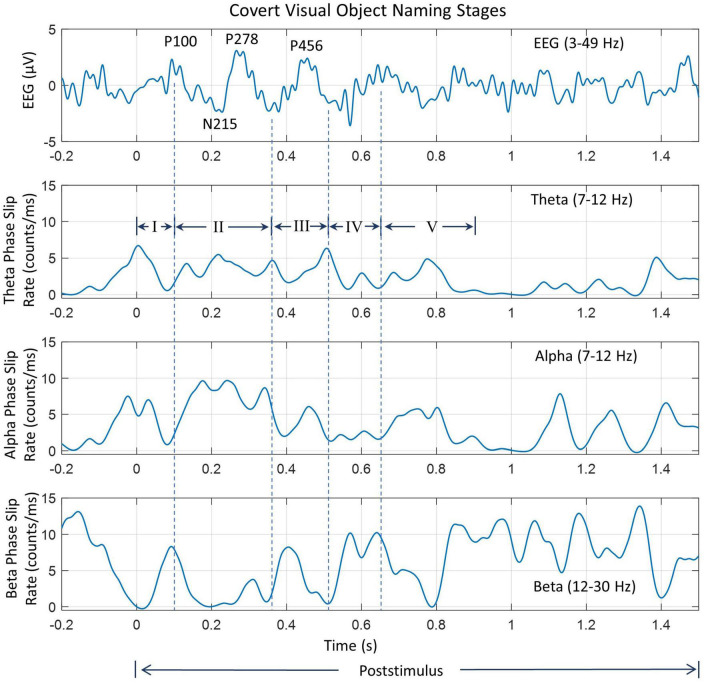
Analysis of insight stages during the post-stimulus period. The **top** plot is the EEG (3–49 Hz) from one of the electrodes in the left visual occipital area, the back of the head. Prominent peaks are marked. The following three plots are for phase slip rates in the theta, alpha, and beta bands, respectively. Vertical dashed lines mark the location of different stages of insight moments. Stage III is the “Eureka” moment.

During the first half seconds of the post-stimulus period, brain activity goes through several stages to capture and recognize the image of the object. As mentioned earlier, these are called insight moments, “Aha” moments, or “Eureka” moments and can be studied during the stimulus period (Kounios et al., 2008) or during the post-stimulus period (Davis et al., 2013; Myers et al., 2014). Our focus will be on the extraction of different stages of insight moments during the post-stimulus period and their durations from the start of the post-stimulus period. There are several stages of this process that are recognizable in the temporal and spatial plots of Figures 4, 6, 7. Our results in Figures 4, 6 show that PSR in the low gamma band is very low in magnitude and very intermittent. In addition, it has been earlier suggested that amplitude modulated phase waves in the theta and alpha bands with a carrier frequency in the beta and gamma bands might be observable in the study of evoked responses (Freeman, 2004; Ruiz et al., 2010; Davis et al., 2013; Kozma and Freeman, 2017). Based on these factors, a choice was made to examine the high temporal resolution behavior of PSRs in the theta, alpha, and beta bands only for the extraction of different stages of insight moments. In Figure 7, time series profiles of the EEG and PSRs in the theta, alpha, and beta bands for one of the electrodes from the left visual area are given. The duration covered is −0.2 to 1.5 s where 0.0 is the start of the post-stimulus period. One should analyze Figures 6, 7 together to get a comprehensive picture of the sequential activation of different regions in theta, alpha, and beta bands during the process of object recognition.

The EEG trace in Figure 7 has several peaks identified during the post-stimulus period. Nomenclature P100 refers to the positive going peak at 100 ms from the start of the post-stimulus period (0.0 s) when the subject intentionally begins to visualize and analyze the name and form, i.e., the shape of the object. Similarly, N215 is the negative going peak at 215 ms. Similarly, other peaks have also been identified. These peaks are different from the P1, N1, and P2 evoked response potential peaks during the stimulus period. Compared to that, the P100, P215, etc., peaks are due to mental imagery, i.e., mental tasks related to object visualization and recognition (Dijkstra et al., 2018; Pearson, 2019).

Some of our results are similar to perception and mental imagery research. The perception is looking at an object which gives rise to VEP P1, N1, and P2 potentials. These relate to the visual cortex getting activated first (∼ 45 ms from the stimulus), and then activity spreads to other parts of the brain, particularly toward frontal and parietal areas for more complex processing to generate a percept, i.e., a mental image of the object after a few 100 s of ms. This relates to the formation of the P2 peak in visual evoked potentials. In our results (Figure 5), this is what we are seeing from the onset of the stimulus. When one visualizes the object during mental imagery, one does not see P1 and N1 peaks of VEP, but rather some activity later on related to complex visual processing and analysis (Dijkstra et al., 2018; Pearson, 2019). In Figure 7, during the post-stimulus period, there is no VEP P1 and N1 peak, but a broad peak between 77 – 215 ms, marked as P100 and N215, which could be considered as related to the mental imagery of the object.

Several vertical dashed lines identify different stages of insight moments. The location of endpoints of different stages was mainly determined from the waveforms and spatiotemporal plots of EEG. This location was then slightly modified based on the waveform patterns of PSRs. This choice was made because the spatiotemporal profiles of EEG were similar from one subject to the other while PSR plots had larger differences in-between subjects. Also, EEG is an independent variable while PSRs are dependent variables derived from EEG. For the Stage I, the visual area (back of the head) is most active between 0.0 ms and 100 ms. The location of the endpoint of Stage I was determined from the location of the peak of the P100 wave which was at 0.1 s for this subject. The location of time points for an overall decline of the PSR activity in the visual area in theta and alpha bands near to P100 wave and the peak of PSR activity in the beta band were determined. A window of ± 20 ms, i.e., between 80 to 120 ms was used for this search. This gives us four nearby time points, one from EEG and the other three from PSRs which were averaged to determine the end point of Stage I.

The endpoint of stage II was determined by searching for the baseline of the P278 wave, i.e., the minimum value between the peaks of P278 and P456. It was at 368 ms from the start of the post-stimulus period. Corresponding to this, the time points for minimum or maximum values of PSR were searched in the period of 0.348 s to 0.388 s. The location of the maximum value of PSR in the theta and alpha bands in the front central area was located. The PSR activity in the beta band is distributed over a large area. The PSR values in the beta band were averaged over all 256 electrodes and the location of the minimum in the window of 0.348 s to 0.388 s was determined. The mean value of these four time points was calculated and that was assigned as the time point for the end of Stage II. Similar procedures were used for determining the endpoints of other stages. A summary of these procedures is given in Table 2. Software codes were written for automatic detection of the end point of all five stages and then manually checked for accuracy and, if needed, corrections were made to the location of the end point of stages.

**TABLE 2:** Determination of the endpoint of insight stages.

Stage	Search interval	Post-stimulus period EEG and PSR search criteria
Stage I	80–120 ms	Location of P100 peak. Time points for decline of alpha and theta PSR, and peak of beta PSR.
Stage II	348–388 ms	Location P278 baseline. Location of overall global maximum of PSR in theta and alpha bands, and minimum of global beta PSR.
Stage III	500–540 ms	Location of P456 baseline. Location of overall global maximum of theta PSR, and minimum of alpha and beta PSR.
Stage IV	630–670 ms	Location of peak around 650 ms. Location of an overall minimum of theta and alpha PSR and the start of the decline of the beta PSR.
Stage V	860–1,000 ms	Start of the resting state EEG. Location of the middle of the quiescent period of theta and alpha PSR, and an overall maximum of beta PSR.

Stage I usually refers to the initial impression of the stimuli on the brain during the post-stimulus period (Mangun, 1995; Schendan and Lucia, 2010). It is also called the ‘Awe’ moment in the knowledge cycle (Myers et al., 2014; Lee et al., 2017). Looking at 0.0 s in Figure 6, the EEG activity is very low, ∼ 1.0 μV in the central area which spreads at 0.1 s to the left and right front temporal, and visual areas where the magnitude is about 3–5 μV. These EEG activity patterns can be related to the P100 wave in object recognition studies during the post-stimulus period. During this stage coordination between different areas of the brain is desynchronized and we see a sudden drop in the phase slips rates in the theta and alpha bands while it is high in the beta band which acts as a carrier wave for the phase slips (Davis et al., 2013; Freeman and Quiroga, 2013; Freeman, 2015).

The spatial patterns of the theta PSR at 0.0 s show medium-level activity (∼ 7 counts/ms) in the frontal area. Refer to Figure 6. After that it shows a focused bright spot in the midline central area at 0.1 s. The spatial pattern of the alpha PSR during this period (0.0–0.1 s) increases in the left parietal, front central, and right centro-temporal areas. During this period, the beta band PSR activity shifts from visual areas to the left frontal and right parietal areas at 0.1 s. This behavior of PSR in theta and alpha bands is very similar to what has been observed and suggested for the behavior of theta and alpha band EEG activities during working memory tasks (Klimesch et al., 2005). During the retrieval of memory, theta EEG behaves like a traveling wave spreading from anterior to posterior sites (Klimesch et al., 2005). When the subject retrieves the image or form of the object in the beginning from the working memory, the theta band PSR becomes widespread in the front central area at 0.0 s and after that, it becomes less and scattered in other parts of the topography during the actual retrieval of the object from the memory. The theta and alpha activity is phase synchronized and linked during working memory tasks (Schack et al., 2005) and this is what we are observing in the behavior of the alpha band PSR.

Stage II (100–370 ms) is characterized by the rapid exploration in the language and memory areas to search for the name and form, i.e., the image of the object. It is identified as the P278 component in Figure 7 during the covert object naming tasks. Stage II stretches to the end of the P278 wave, approximately about 370 ms. During the Sage II, there is strong coordination in different areas of the brain, particularly between language and memory areas. This stage is commonly called the “Incubation” stage in creative thinking literature. This can be observed in Figures 6, 7. The PSR activity in the theta band is very high in the language and memory areas, near the midline central, at 0.3 s. The interconnected parietal activity area is also recognizable in the PSR activity in the alpha and beta bands. This stage is often characterized by N300 and P300 waves in the literature and plays an important role in object recognition and emotional responses to the stimuli (Carretié et al., 1997; Sprugnoli et al., 2017; Kumar et al., 2021).

Stage III (370–520 ms) is the “Eureka” moment, also called the “Illumination” moment, when one recognizes and identifies the object (Sadler-Smith, 2015). The endpoint of this stage was determined by the location of the PSR peak in the theta band, and the baseline in the alpha and beta bands in Figure 7. In addition, their spatial plots in Figure 6 were also used to determine the appropriate endpoint of Stage III. During this period, the activity is mainly in the language area, i.e., left centro-parietal areas, with interrelated modulated activities in the frontal and left parietal areas in the theta band (Figure 6), central and left parietal areas in the alpha band (Figure 6 at 0.4 and 0.5 s), and several hot spots in frontal, right parietal and left central areas in the beta band (Figure 6 at 0.4 s). There are noticeable differences between the PSR and EEG spatial plots suggesting that the coordinated activity of cortical neurons is in some other areas of the brain.

Stage IV (520–650 ms), called the “Verification” stage (Davis et al., 2013; Sadler-Smith, 2015) is the integration of the new knowledge in the memory for immediate future use, such as, in covert or overt visual object naming tasks. The EEG activity begins to spread in all parts of the scalp topography (Figure 6, 0.5–0.7 s, top row), while the PSR activity in the theta band becomes less and less during this period. The PSR activity in the alpha band also exhibits a general spreading from posterior to frontal areas of the topography with heightened focused activity in the left parietal and midline front central areas. This suggests that the parietal and frontal area coupling (Jung and Haier, 2007) continues from Stage III to Stage IV during the object recognition and decision-making processes in the brain. The endpoint of Stage IV was estimated where the PSR activity was near the minimum in the theta and alpha bands while the EEG magnitude was highest.

Stage V (650–880 ms) is the start of the return to the background activity of the brain. The endpoint is difficult to ascertain; it could be anywhere between 0.880 s to 1.0 s based on the spatial and time series plots of PSR in different bands. During this stage, the PSR activity in the theta band spreads from left central to parietal areas and then in the whole topography at 0.7 s and after that settles down in the frontal area. The alpha band PSR activity shifts from the central midline to the left parietal (0.7 s), then to the frontal areas at 0.9 s. The beta band PSR activity remains very strong in the central and parietal areas of the brain during this stage. There are significant differences between the EEG and PSR plots during this stage.

A paired Student’s *t*-test analysis was performed on the mean values of PSR for different stages of insight moments. The baseline values were selected from the end of the stimulus period between −0.7 to 0.0 s. The duration of each stage is different. So, the baseline was selected of the same length. We get 256 values, one for each electrode for the baseline and each insight stage. The paired Student’s *t*-test was performed on the baseline and values of each insight stage. This was performed for each band, theta, alpha, beta, and low gamma bands, separately. It was found that baseline and insight stage values were significantly (*p* < 0.05) different for each insight stage in all four bands.

### 3.2. Combined results of five subjects

#### 3.2.1. Data averaging

**FIGURE 8:**
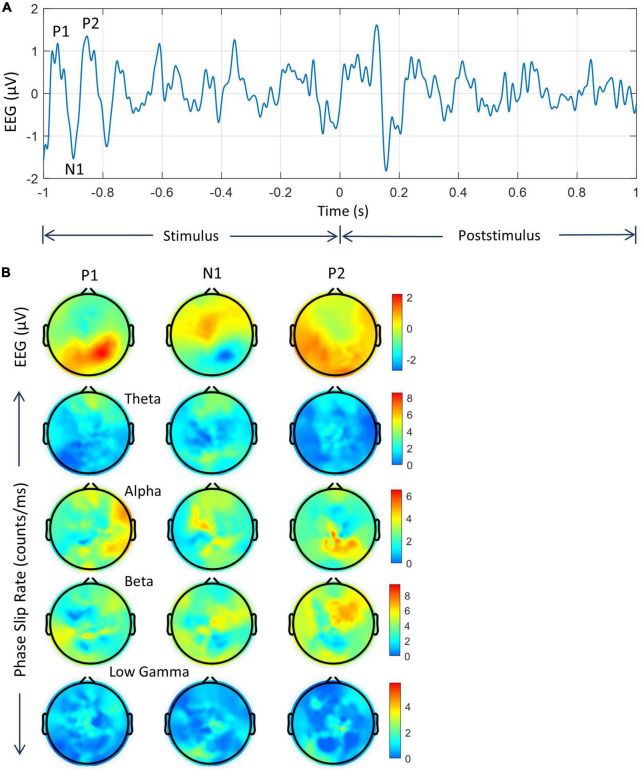
Analysis of averaged data of five subjects during the stimulus period. **(A)** EEG trace from one of the electrodes in the left visual area. P1, N1, and P2 peaks are marked. **(B)** The top row is for the spatial plots of EEG in the 3–49 Hz band, followed by phase slip rates in all four bands.

**FIGURE 9:**
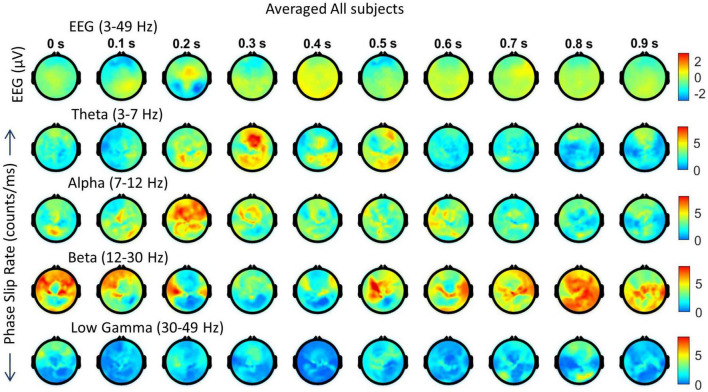
Spatiotemporal plots of the averaged data of five subjects. **(Top row)** EEG (μV) in the 3–49 Hz band. This is followed by PSRs (counts/ms) in theta, alpha, beta, and low gamma bands. The temporal separation between frames is 100 ms.

As stated earlier, the EEG data were averaged over 29 trials for each subject. A similar analysis was performed on the remaining four subjects. The PSR values for each subject were computed from their respective EEGs which were averaged over 29 trials. Spatiotemporal plots of the results of all five subjects were similar but with noticeable individual differences due to the differences in latencies of evoked responses. It will be overwhelming to include plots for all five subjects. Thus, for plotting purposes only, first, for each subject, the EEG data were averaged over 29 trials and then the trial-averaged data of five subjects were averaged. Similarly, the computed PSRs of all five subjects were averaged and their spatiotemporal plots during the stimulus and post-stimulus periods are given in Figures 8, 9, respectively. Also, averaging reduces the background variations while enhancing common features present in all subjects. The averaged values of the different stages of insight moments for all five subjects are also included.

#### 3.2.2. Stimulus period of averaged data

Analysis of the averaged EEG in 3–49 Hz band and averaged PSR data for each band during the stimulus period is given in Figure 8. The time series of EEG (3–49 Hz) data of one of the electrodes in the left visual area is given in Figure 8A for the duration of −1.0 to + 1.0 s. The location of P1, N1, and P2 peaks during the stimulus period is at 45 ms, 101 ms, and 161 ms, respectively from the start of the stimulus at −1.0 s. These locations of averaged peaks are slightly different from subject #1 as given in Figures 4, 5 because of the differences in latencies of the five subjects.

The spatial plots for P1, N1, and P2 are given in Figure 8B. The positive EEG activity at the P1 peak is mainly in the visual (back of the head) area, at the N1 peak is in the front central areas, and at the P2 peak is widespread in the visual areas, and left and right temporal areas. The negative N1 activity is the right visual and parietal areas. In a partial way it matches with the spatial plots of subject #1 in Figure 5. The theta band PSR at the P1 peak is widespread in the frontal area, at the N1 peak in the frontal and the visual areas, and at the P2 peak in the midline areas. The alpha band PSR at the P1 peak has strong activity in the frontal, visual, and right temporal areas, at the N1 peak in the left central and midline areas, and at the P2 peak in the right midline parietal areas. The beta band PSR at the P1 peak is in the left parietal and right frontal areas, at the N1 peak in the right central and visual areas, and at the P2 peak in the right frontal areas and also distributed in the left temporal areas. The PSR in the low gamma band is of low (1–3 counts/ms) intensity distributed all over the scalp. The PSR plots are different from the EEG plots and this suggests that PSR activity picks up different activity areas in the brain.

Similar to Subject #1, an ANOVA analysis was performed on PSR values at P1, N1, P2, and the values at the beginning of the stimulus period. This was performed for each band separately. It was found that values for each band were significantly different (*p* < 0.01) from each other.

#### 3.2.3. Averaged spatial plots during post-stimulus period

These plots are given in Figure 9. The spatial patterns of EEG are similar to the first subject but there are noticeable differences. Between 0.0 to 0.3 s, the EEG activity patterns shift from the midline to the parietal, then to the frontal midline (0.2 s), and then back to visual areas at 0.3 s. These correspond to the P100, N215, and P278 waves (Figure 7) during the visual object naming tasks. After that, the EEG activity is distributed in wide areas at 0.4 s, then gradually spreads in posterior areas at 0.6, then right frontal areas at 0.7 s, and then after that in other parts of the topography. The PSR activity in the theta band is very strong in the visual areas at 0.2 s and then gradually shifts from frontal to right parietal areas during 0.3–0.5 s. After that, at a low level (∼ 0–5 counts/ms) it spreads to posterior and frontal areas during 0.5 to 0.9 s. The PSR activity in the alpha band has good similarities with EEG activity at the beginning between 0.0 to 0.2 s and then some similarities later on between 0.5 to 0.9 s. The PSR activity in the beta band is very strong with visible spatiotemporal waves over the 0.0 to 0.9 s period. The activity shifts from frontal to parietal areas over the course of time. Similarly, the PSR in the low gamma band shifts from frontal to parietal, then to frontal, and then spreads in a wide area at 0.8 s. Similar to the beta band, spatiotemporal wave patterns are also present in the low gamma band.

A paired student’s *t*-test analysis was performed on the averaged data sets. It was performed on the mean values of PSR for the 1-s-long data during the stimulus (−1.0 to 0.0 s) period and the same length (0.0 to + 1.0 s) of data during the post-stimulus period. The PSR values were averaged over the time domain only. Thus, there were 256 values, one for each electrode, for the stimulus and post-stimulus period, respectively. This was performed for each band separately. Pre and post-stimulus values were significantly (*p* < 0.01) different for each band.

#### 3.2.4. Averaged duration of insight moments

The duration of different insight stages for all five subjects was computed based on the procedures described for subject #1. These values are listed in Table 3.

**TABLE 3:** Duration of different stages of insight moment.

	Stage I	Stage II	Stage III	Stage IV	Stage V	Total
Subject 1	100	270	150	130	230	880
Subject 2	96	260	170	168	210	904
Subject 3	70	240	228	156	205	899
Subject 4	81	231	145	202	220	879
Subject 5	105	253	165	140	200	860
Mean ± std	90 ± 14	250 ± 15	172 ± 33	159 ± 28	213 ± 12	884 ± 18

All values are in milliseconds and mean ± std values have been rounded off to the nearest millisecond.

These durations of different stages in visual object recognition and naming tasks are within the limits of previous studies for the post-stimulus period (Davis et al., 2013) and could provide some correlations with previous insight moment studies for the stimulus period (Kounios et al., 2008; Daliri et al., 2013). The combined duration of Stage I and II will give us a time frame of approximately 319 ± 9 ms which will match well with the P3 (or P300) wave to recognize objects in visual object recognition experiments (Johnson and Olshausen, 2003). These similarities of time frames might be of interest to relate cortical phase transitions to the P3 wave in object recognition processes. Overall, for the Eureka effect, the combined duration of Stage I to Stage III is about 512 ± 21 ms which is within the range of earlier reported results derived from the stimulus period alone (Daliri et al., 2013; Kounios and Beeman, 2014; Sprugnoli et al., 2017). Summing up the duration of all five stages, one will get a time frame of approximately 884 ± 18 ms to recognize, identify, and assimilate the new knowledge and then start the return to the normal background mental processes takes place. One could call this the duration of a complete mental task, particularly for the covert visual object naming tasks during the post-stimulus period.

## 4. Discussion

Our objective was to examine how phase transitions of the coordinated activity of cortical neurons behave during visual object naming tasks. Our results show that phase slips and phase slip rates (PSRs) complement to what one sees from EEG. Often the phase slip activity is observed in different areas as compared to the spatial patterns of EEG. This indicates that a combined analysis with spatial plots of EEG and PSR gives us a comprehensive picture of the underlying brain activity. As suggested earlier that two processes are going on which contribute to the measured EEG. One is the random firing of cortical neurons which gives rise to the bulk of measured EEG. The other is due to the phase transitions of coordinated activity of cortical neurons at microscopic and mesoscopic scales that produce small perturbations in the bulk of the EEG data. In traditional EEG analysis, such as power spectral density (PSD) computations, these small perturbations contribute very little and remain hidden within the PSD of the bulk of the EEG. To separate and extract the contributions of these small perturbations one has to use the Hilbert transform which adds ± π phase shift at each small perturbation. This makes it easier to extract the phase slips related to the phase transitions of the coordinated activity of cortical neurons. Our results indicate these procedures work and we were able to study the phase slip activity from different parts of the brain during the stimulus and post-stimulus periods. In general, the coordinated activities of cortical neurons happen at mesoscopic (∼ 0.5 mm) scales in the cortex and one can see the related phase slips over an area of a few centimeters of the scalp surface which could be easily visible on four to six nearby electrodes (Freeman, 2015). If larger areas of the cortex go into the criticality, then one will see these phase slips distributed over large areas of the scalp surface. An example of this will be in PSR activity (Figure 9) in the beta band between 0.5 s to 0.8 s. The 0.5 s spatial plot is in Stage III, the ‘Eureka’ moment, and the 0.6 s plot is in Stage IV, the ‘Verification’ stage of the insight moments. During both of these stages, one would expect that different areas of the brain are very active to recognize and verify the object which will produce phase slip activity in a larger area. We believe this is what we are seeing in some of our spatial plots.

Our results show that one can study the temporal sequence of the phase slips and related cortical phase transition states during the object naming tasks from the scalp EEG data and this might be a new promising way to study cortical neurodynamics. These durations of different stages of insight moments described above are very similar to earlier studies related to phase cone formations in ECoG data of rabbits during conditioned visual stimuli experiments (Davis et al., 2013; Myers et al., 2014). Also, peaks and valleys of the oscillatory patterns of phase slips match with the peaks of P1, N1, and P2 waveforms of visual evoked potentials during the stimulus period in human EEG (Kounios et al., 2008; Simanova et al., 2010; Woodman, 2010; Daliri et al., 2013; Sarathy, 2018) and also for the object naming tasks during the post-stimulus period (Davis et al., 2013; Myers et al., 2014). Similar results were also observed from the ECoG data of epilepsy patients during visual evoked studies (Liu et al., 2009) which confirms what we see in our results. Overall, as shown that this phase slip technology is a new complimentary tool and, possibly, it could have wider applications to study the cortical phase transitions from the resting state and evoked potential EEGs. However, our findings need to be examined with data from more subjects before they could be useful in a reliable fashion.

The underlying structure below the theta and alpha phase waves could be related to the coordinated activity of cortical neurons at mesoscopic (∼ 0.5 mm) or sub-mesoscopic (∼ 0.1–0.5 mm) scales. The physiological basis could be the dendritic currents and the speed of propagation (1–10 mm/ms) on cortical fibers at mesoscopic scales. However, these concepts need to be investigated through simulations with neural mass models or measurements from cultured neuronal preparations in Petri dishes.

After the stimulus is turned off, the VEP still exist (Marcar and Jäncke, 2016) which could compromise the potentials seen in visual imagery. Carefully designed studies are needed to separate the visual evoked potentials and the potentials arising due to the mental imagery. These studies need to examine the duration of the stimulus period, the delay between stimulus-off and the start of the mental imagery period, and the effects of any audio or visual cue given to start the mental imagery period because these cues could also start some cognitive processes in the brain. Source reconstructions will be the one way to see which brain areas are active at a given time point to solve this puzzle. In this approach, one needs to use anatomically realistic human head models that include the dura layer to reduce localization errors (Ramon et al., 2006, 2014). Another approach might be to use the cortical phase transition techniques described here to differentiate between perception and mental imagery.

## Disclosure

Some initial findings were presented at the IAN 2019, New Delhi, XXXVII Annual Meeting of Indian Academy of Neurosciences, 18–21 November 2019, New Delhi, India.

## Data availability statement

The raw data supporting the conclusions of this article will be made available by the authors, without undue reservation.

## Ethics statement

The studies involving human participants were reviewed and approved by the Ethics Committee of Reykjavik University, Iceland. The patients/participants provided their written informed consent to participate in this study.

## Author contributions

All authors contributed equally in the design of the experiment, data collection, data analysis, and manuscript preparation.
